# Intra-V1 functional networks and classification of observed stimuli

**DOI:** 10.3389/fninf.2024.1080173

**Published:** 2024-03-11

**Authors:** Marlis Ontivero-Ortega, Jorge Iglesias-Fuster, Jhoanna Perez-Hidalgo, Daniele Marinazzo, Mitchell Valdes-Sosa, Pedro Valdes-Sosa

**Affiliations:** ^1^The Clinical Hospital of Chengdu Brain Sciences, University of Electronic Sciences Technology of China, Chengdu, China; ^2^Cuban Center for Neuroscience, Havana, Cuba; ^3^Department of Data Analysis, Ghent University, Ghent, Belgium

**Keywords:** V1, fMRI, functional networks, SVM-classifier, Navon task, weight-maps

## Abstract

**Introduction:**

Previous studies suggest that co-fluctuations in neural activity within V1 (measured with fMRI) carry information about observed stimuli, potentially reflecting various cognitive mechanisms. This study explores the neural sources shaping this information by using different fMRI preprocessing methods. The common response to stimuli shared by all individuals can be emphasized by using inter-subject correlations or de-emphasized by deconvolving the fMRI with hemodynamic response functions (HRFs) before calculating the correlations. The latter approach shifts the balance towards participant-idiosyncratic activity.

**Methods:**

Here, we used multivariate pattern analysis of intra-V1 correlation matrices to predict the Level or Shape of observed Navon letters employing the types of correlations described above. We assessed accuracy in inter-subject prediction of specific conjunctions of properties, and attempted intra-subject cross-classification of stimulus properties (i.e., prediction of one feature despite changes in the other). Weight maps from successful classifiers were projected onto the visual field. A control experiment investigated eye-movement patterns during stimuli presentation.

**Results:**

All inter-subject classifiers accurately predicted the Level and Shape of specific observed stimuli. However, successful intra-subject cross-classification was achieved only for stimulus Level, but not Shape, regardless of preprocessing scheme. Weight maps for successful Level classification differed between inter-subject correlations and deconvolved correlations. The latter revealed asymmetries in visual field link strength that corresponded to known perceptual asymmetries. Post-hoc measurement of eyeball fMRI signals did not find differences in gaze between stimulus conditions, and a control experiment (with derived simulations) also suggested that eye movements do not explain the stimulus-related changes in V1 topology.

**Discussion:**

Our findings indicate that both inter-subject common responses and participant-specific activity contribute to the information in intra-V1 co-fluctuations, albeit through distinct sub-networks. Deconvolution, that enhances subject-specific activity, highlighted interhemispheric links for Global stimuli. Further exploration of intra-V1 networks promises insights into the neural basis of attention and perceptual organization.

## Introduction

**Figure 1:**
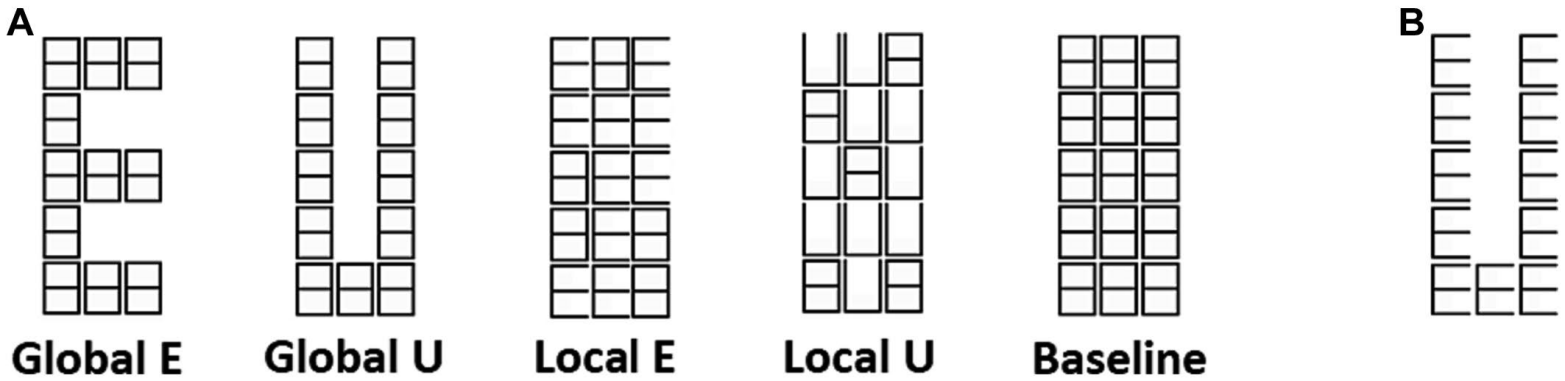
Navon figures. **(A)** Two letters (E and U), and two levels (Global and Local) of modified Navon figures used in the experiment. **(B)** An example of traditional Navon figure.

Two prior studies using functional magnetic resonance imaging (fMRI) have demonstrated that BOLD activity from different sites in visual striate cortex (V1) exhibits stronger correlations when mapping parts of a unique visual object, as opposed to pieces of distinct objects (Nasr et al., 2021; Valdes-Sosa et al., 2022). These network effects may reflect various cognitive mechanisms, such as perceptual organization and object-based attention at very early stages of visual processing and deserve further study. Here, we address four issues: (1) Do intra-V1 correlations carry multivariate information about observed stimuli, and is this information stable across individuals? (2) Is information about more abstract properties of a visual property (tolerant to changes in another property) present in the intra-V1 correlations? (3) Is it possible to narrow down the physiological sources shaping the information present in intra-V1 correlations? 4) Are the intra-V1 patterns of synchrony driven by eye movements? To answer these questions, we reanalyzed fMRI data from our previous article (Valdes-Sosa et al., 2022) obtained while observers monitored modified Navon figures (Iglesias-Fuster et al., 2014) consisting of the two letters E and U, each presented at a Global or Local level (Figure 1).

In these new analyses we used multivariate pattern analysis (MVPA) of the intra-V1 correlation matrices. This departs from the prior studies of intra-V1 synchrony which used univariate statistical tests. Univariate tests may overlook systematic associations between features, potentially missing more complex patterns (Haynes and Rees, 2006; Davis and Poldrack, 2013). The univariate approach is also uninformative about the stability of network topologies across individuals, which is crucial when studying a small number of participants (Adali and Calhoun, 2022). These limitations can be circumvented with MVPA.

We first employed inter-subject MVPA (Kaplan and Meyer, 2012; Rice et al., 2014; Ramírez et al., 2020; Wang et al., 2020) to see if it was possible to predict the properties of stimuli (defined by the conjunction of level and shape) presented in the experiment from the intra-V1 correlation matrices and to assess the stability across individuals of the overall network topologies associated with these stimuli. We dub these classifications here as ‘specific’. Note that inter-subject MVPA critically depends on the ability of anatomical normalization to align spatially structured neural patterns across individual brain, and on the granularity of the spatial units comprising the fMRI signals (Ramírez et al., 2020). This alignment may fail even when using normalization based on cortical surface features (Robinson et al., 2014), although, it is still superior to volume based alignment (Hagler et al., 2006). However, the correspondence of V1 retinotopic mapping with surface landmarks is very tight across individuals (Benson et al., 2012; Benson and Winawer, 2018), which makes this area an excellent focus for inter-subject MVPA.

Related to the second question, it is crucial to recognize that intra-V1 correlations may carry two types of stimulus-related information: one tied to the specific retinotopic pattern of individual stimuli (the conjunctions mentioned above) and the other linked to more abstract properties. Therefore, we also trained intra-subject (and inter-subject) cross-classifiers (Kaplan et al., 2015; Hebart and Baker, 2018) to predict one property (level or letter shape) in the presence of a shift in the other property. Successful cross-classifiers in this case could suggest that neural representations related to one attribute are tolerant to changes in the other attribute, and are more ‘abstract’ than those coding specific conjunctions of features.

**Figure 2:**
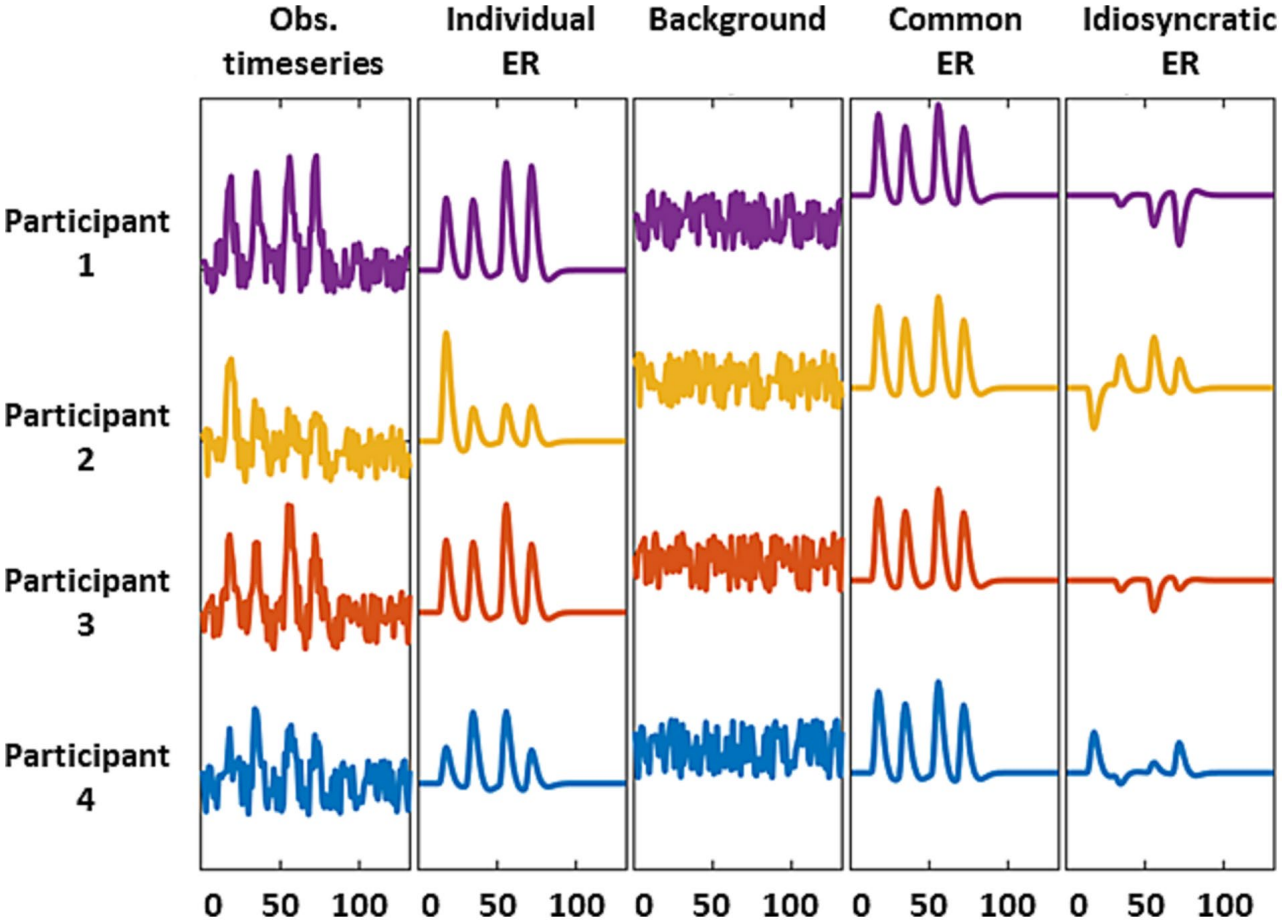
Schematic representation of the fMRI source models used here. We considered observed signals = individual stimulus-evoked responses (ERs) + background activity (first three columns). The common (or shared) response is the average of the individual ERs and suppresses background and idiosyncratic ERs (column 4). The residual activity after deconvolving with a GLM retains the background and idiosyncratic ERs due to mismodelling of the individual ERs.

The third question arises because several neural sources, including stimulus-evoked responses and background activity, may shape the information carried by intra-V1 correlations. Stimulus evoked activity may be shared across, or be idiosyncratic to, individuals, whereas background activity is always idiosyncratic (discussed in Nastase et al., 2019). Although these contributions are mixed in the fMRI signal, it is possible to emphasize or suppress some components through appropriate preprocessing (Figure 2). Stimulus-evoked responses shared by all individuals, which we dub as the common response (CR), can be emphasized by averaging across participants which enables studying inter-subject correlations. They can also be de-emphasized by deconvolving the fMRI data in specific individuals with hemodynamic response functions (HRFc) or finite impulse responses (FIR) using general linear models (GLM). Deconvolution shifts the balance towards participant-idiosyncratic activity (see Figure 2). Consequently, we examined the performance of the classifiers -based on intra-V1 synchrony- when stimulus-evoked common responses were favored or suppressed by the preprocessing schemes explained above.

Expanding on the previous ideas, distinct neural sources contributing to intra-V1 synchrony can influence the accurate prediction of stimulus properties, in our design, based on different features. Remember, that the features here are intra-V1 network edges, in other words the strength of functional connections between two points on V1. Since each V1 site has a direct mapping to the visual field measured in angles from fixation, each edge can be described also as the connection between two sites in the stimulus plane. Thus, each link’s weight (strength) can be plotted in the visual field as the thickness of the line connecting two cortical sites or measured in subsets of VF networks. Here, V1 sub-networks were defined as the connections between the V1 regions that mapped different visual field quadrants. Thus, each sub-network consisted of all the links between V1 nodes mapping one quadrant of the visual field on the one hand and nodes mapping another quadrant on the other.

The contribution of V1 sub-networks to each of the classification tests can be gauged by the feature weight maps underlying successful stimulus predictions. These maps are simply the values of the estimated coefficients in the discrimination equation (here one for each intra-V1 connection). After training, the classifier equation assigns distinct coefficients to network links (the features). This equation yields a linear combination of features from new data to predict the associated stimulus class. These coefficients, called classifier weights, are commonly used to gauge feature importance in classification. Since successful classification indicates the presence of information in a cortical region, features with the absolute largest weights are sometimes interpreted as carrying most of the information that the researcher (but not necessarily the brain) is decoding. However, interpreting large weights as indicative of information strength for a cortical feature can be misleading. While significant weights suggest signal presence, their magnitude may also denote their role in mitigating irrelevant noise. Classifiers function as backward extraction filters, detecting the signals guiding classification and filtering out noise.

Haufe et al. (2014) proposed a transformation that converts backward filters into approximations of activation patterns in a corresponding forward model. The activation (or, in our case, connection) patterns are latent (hidden) factors that can be inferred from the observed weights by the transformation (see Haufe et al., 2014, for details of this operation) and are better suited for functional interpretation since they reflect less the operation of filtering noise and more the presence of signals. Therefore, instead of the raw feature weight maps, we used the framework of ‘forward models’ described above to transform weight maps into activation maps. Despite its limitations (Douglas and Anderson, 2017), this transformation enhances the interpretability of brain predictive patterns. The sign of a feature weight indicates which of the classes it supports in a binary classification. Therefore sub-networks can be further segregated according to the class they support in addition to the quadrants that they connect. The consistent mapping of the visual field (VF) onto V1 simplifies understanding of these weight maps (Benson et al., 2012; Benson and Winawer, 2018).

The final question is related to another potential source of intra-V1 fMRI synchrony: eye movements. Gaze displacements over the stimuli could change the parts of stimuli activating each V1 site thus affecting the fMRI signal. If these displacements change across stimuli, they will generate different patterns of intra-V1 synchronization. Previous studies have found highly divergent gaze patterns when attending to the Global and Local levels of Navon stimuli (Sasaki et al., 2001). Thus, bursts of fMRI activity triggered by fixations on different stimulus parts could generate intra-V1 correlations that differ markedly between the Global and Local conditions, for example. To control for this possibility, we performed a post-hoc analyses of gaze patterns using the fMRI signal of the eyeballs (Frey et al., 2021) from the original experiment. We also measured the eye movement patterns for each of our four stimuli in an offline control experiment and simulated the potential effects of the measured eye movements on the fMRI activity and intra-V1 correlations to see if this could explain classifier success.

## Materials and methods

The fMRI data used here is described in other publications (see Valdés-Sosa et al., 2020, 2022, for more details), and a summary description is provided below.

### Participants

Twenty-six human volunteers (ages 23 to 28 years; 9 females) participated in the study. All had normal, or corrected-to-normal, vision, did not present any medical condition, and were right-handed except for two cases. The procedures were approved by the ethics committees of the University for Electronic Science and Technology of China (UESTC) and the Cuban Center for Neuroscience. Participants gave written informed consent in compliance with the Helsinki Declaration.

### Stimuli and tasks

Modified Navon figures (Figure 1) were presented in the experiment. Two letters, E and U, each at the Global and Local levels, were used in a blocked stimulus design. A background matrix was made from white lines on a black background and was about 2.0 degrees of visual angle (DVA) wide and 5.3 DVA high. The overall matrix was built out of smaller placeholder elements shaped like ‘8’s (each with about 40 min of DVA wide and 1 DVA and 3 min high). Only one letter type (unveiled by erasing some lines in the matrix) was shown in each block and was repeatedly presented for 1 s (alternating with the background also flashed for 1 s). The participants were required to report the number of minor deviations in letter shape in each block. The stimuli were projected on a screen at the subject’s feet, viewed through an angled mirror fixed to the MRI head coil, and were generated using the Cogent Matlab toolbox.^1^

Blocks had 44 s of duration and consisted of an initial cue (‘Global’ or ‘Local’) presented for 1 s, followed by a 19 s baseline, followed by 20 s letter repetitions, and ended with a 4 s wait period where the number of shape deviations was reported. Five runs were presented in 24 participants and four runs in two, each consisting of 12 blocks (3 blocks for each letter: EG, EL, UG, and UL).

### Data acquisition and image preprocessing for the main experiment

Recordings were carried out with a GE Discovery MR750 3 T scanner (General Electric Medical Systems, Milwaukee, WI, United States) using an eight-channel receiver head coil. Functional images were obtained with a T2*- weighted echo planar imaging sequence (TR = 2.5 s; TE = 40 ms; flip angle = 90^○^) with a spatial resolution of 1.875 × 1.875 × 2.9 and 135 images per run. A T1-weighted image was also obtained with 1 × 1 × 0.5 mm resolution.

Initial pre-preprocessing of functional data included discarding the first five volumes of fMRI in all runs, artifact correction using ArtRepair toolbox^2^, followed by slice-timing, head motion correction (with the extraction of motion parameters) and unwarping with SPM8.^3^ The T1 Image was segmented and normalized to MNI space using SPM12 to extract nuisance parameters from fMRI activity in white matter (WM) and cerebrospinal fluid (CSF) that were included in the general linear model (GLM) described below. For each subject, these masks were created using a threshold of tissue probability greater than 0.9. The CSF mask was also restricted to the ventricles using a template in MNI space.^4^

Cortical surfaces (white and pial) were reconstructed from the T1 Image for each subject using Freesurfer,^5^ were registered to the FsAverage template, and subsampled to 81,924 vertices. The mid-gray cortical surface was co-registered with the functional data, and then the fMRI time series were interpolated to each mid-gray cortical surface. Here, only time series for V1 were studied. In a few cases, data was missing from some V1 vertices due to noise or BOLD signal dropout at specific cortical vertices, which were concentrated in small areas of the V1 visual field (see Valdes-Sosa et al., 2022). The functional data were converted to Cifti files, and the *-cifti-dilate* command from HCP workbench software^6^ was applied to impute the missing data. Missing vertex values were replaced by a distance-weighted average of nearby good values, but only if the missing value neighbored or was within 7 mm of the geodesic distance of a valid value. Then the *-cifti-smoothing* workbench command was applied (using a Gaussian kernel of 2 mm) to smooth the functional data over the surface to reduce random noise in the signal.

Finally, to additionally suppress the effects of noise, artifacts, and physiological contaminants, the data was high pass filtered with a time constant of 128 s, and the effect of 64 nuisance parameters was regressed out by applying a general linear model (GLM). The nuisance regressors included the primary motion parameters, their derivatives, and the quadratics of both these sets (24 motion regressors in total). Also, physiologic noise was modeled using the aCompCor method (Behzadi et al., 2007) on the time series extracted separately from the masks of WM and CSF in ventricles in volume space. The first five principal components from each set of time series, the derivatives of these components, and the quadratics of all these parameters were obtained (40 regressors in total). After noise regressing, each surface vertex’s residual time series was submitted to different preprocessing schemes to generate three types of intra-V1 connectivity matrices (described below).

### Estimation of fMRI connectivity matrices based on different neural sources

Only data from the V1 region representing the central 4 DVA of eccentricity defined with probabilistic eccentricity and visual region maps^7^ were analyzed. For all preprocessing schemes, the time series were segmented into blocks corresponding to stimulus presentations (adjusting for the time shift introduced by the hemodynamic lag). These segments were linearly detrended, and segments corresponding to the same stimulus were concatenated, which yielded 120 points (equal to 300 s) for each stimulus type in 22 participants and about 96 points (equal to 237.5 s) in another four subjects. Prior studies find that resting state fMRI analyses with concatenated data are not significantly different from those with continuous data in multiple aspects (Zhu et al., 2017; Cho et al., 2021). Intra-V1 connectivity matrices were estimated in all participants by calculating the Pearson correlation coefficient between all V1 vertices segregated by stimulus condition (EG, EL, UG, and UL).

The Common Response matrices (dubbed CR) were estimated by the correlation between one individual V1 time series and the grand average time series for the other individuals (see Nastase et al., 2019). This correlation was calculated in both directions ( $\mathrm{corr}\left(S_{i},S_{n-1}\right)$ and $\mathrm{corr}\left(S_{n-1},S_{i}\right)$ ) and averaged to obtain a symmetric correlation matrix. This procedure suppressed participant idiosyncratic activity and residual noise (Figure 2). Additionally, two different GLMs were employed (before detrending the time segments) to suppress common activity and relatively enhance participant-idiosyncratic activity. In one case, the stimulus responses for each block were modeled as a square wave convolved with the canonical hemodynamic (HRFc). In the other case, finite impulse response (FIR) functions were modeled with nineteen Dirac deltas with unit amplitude. These two HRF models were adopted from SPM 12. After deconvolving the response to the stimulus in these GLMs, the residual time series were used to estimate the connectivity matrices.

Comparing classification accuracy after different fMRI preprocessing schemes allows assessment the relative contribution of stimulus-evoked activity and background activity to intra-V1 correlations. One cannot assume that deconvolving eliminates the stimulus-all evoked activation since HRF modeling is likely imperfect. However, if classification is not affected or improves after the reduction of the evoked response via GLM, background activity could play a more significant role in shaping the classifications. Note that mis-modeling of the HRF should be smaller (although not disappear) with the flexible FIR model which fits a different HRF for each stimulus type (Lindquist et al., 2009). Finally, for the classification analysis, all matrices were vectorized. The correlation values were converted to *z*-values using the Fisher r to z transformation. Negative values in the matrices were set to zero, since positive and negative correlations (the latter anti-correlations) define different brain networks (e.g., Uddin et al., 2009). However, the use of negative correlations has been questioned (Buckner et al., 2013). We preferred to avoid the debate, although negative correlations should be explored in future related work.

### Two types of MVPA

Inter-subject specific classification (to assess stability across participants) and within-subject abstract cross-classification (to assess discrimination invariance) were performed, in which the accuracy in predicting observed stimuli from the intra-V1 connectivity matrices was measured. The connection strengths between all node pairs were used as features in all tests, and a support vector machine (SVM) was employed as the classifier (using a lineal kernel ( $G\left(x_{i},x_{j}\right)=x_{i}\prime x_{j}$ ) and the default parameter C = 1). Feature selection was performed to eliminate less relevant connections by applying a two-tailed t-test for each feature between conditions across the participants in the training data. Only links with significant *t*-values (*p* < 0.01) were retained. Prediction accuracy was used to assess the performance of each classifier, and the statistical significance of deviation from a random classification (0.5 correct) was estimated by permutation testing (1,000 times), in which stimulus labels were randomly changed (Valente et al., 2021). The accuracy of the two instances of each type of classifier was averaged, and the probabilities of the associated permutation tests were combined with the Fisher formula (Fisher, 1925).

### Inter-subject classification tests

Inter-subject classification tests were performed to evaluate if the pattern of association between specific stimulus conditions and V1 network topology was stable across participants. These tests were carried out with cross-validation in a leave-one-subject-out (LOSO). Thus, training was based on the data of n-1 participants and testing on the data of the left-out participant. The four discriminations tested were level (Global vs. Local), separately for the E and the U stimuli, and letter shape (E vs. U), separately for the Global and the Local stimuli. Every iteration of the LOSO consisted of 50 training and two testing samples.

### Intra-subject cross-classification tests

Cross-classification tests were employed to see if models built for a relevant feature (e.g., level) were invariant to changes in another irrelevant feature (e.g., letter identity). The data from all subjects were divided into two sets of pairs (each with 52 observations) to test the invariance of level discrimination with respect to changes in letter identity and the invariance of letter discrimination with respect to changes in level. The classifier was based on the EG vs. EL, and UG vs. UL pairs for abstract Level. The classifier was based on the EG-UG and EL-UL pairs for abstract Letter. The classifier was trained twice, alternating which pair was used for training and which for testing, and the two accuracies were averaged. Note that in these tests cross-validation is not needed since the classifier was trained with data from one pair of conditions and tested with independent data related to the other. In this case, permutation tests were based on randomizing the labels only of the training data in both directions of the test. However, to see the reliability of the abstract cross-classification we also performed the inter-subject approach described above.

### Analysis of weight maps in the cross-classification tests

The weight maps of the SVM in the abstract Level cross-classifier were examined to determine which connections within V1 contributed most to classification performance for the different preprocessing schemes (CR, HRFc, and FIR). This analysis was not carried out for the abstract Letter cross-classification for reasons explained below. This analysis was also not performed for the specific inter-subject tests since their weight maps necessarily diverge because they reflect the retinotopic stimulation pattern. In contrast, the invariance of weight maps for one attribute despite changes in another implies a generalization beyond the precise layout of retinotopic stimulation.

To obtain more robust summaries of the weight maps and assess their stability a bootstrap (1,000 times) procedure was performed. In each bootstrap iteration, resampling with replacement across participants was carried out. The SVM was then re-trained in each replication to obtain multiple models for classifying level for both E and U letters. The estimated model coefficients were transformed into forward models (Haufe et al., 2014), producing transformed weight maps to enhance interpretability.

The concordance of transformed weight maps rankings between the two instances of the invariant cross-classifications was measured. If cross-classifiers trained separately for two directions yield similar weight maps, then the features guiding the generalization of learning across the two situations are equivalent. This is a sign of an abstract representation. Concordance was measured between the two instances for each bootstrap replicate using Kendall’s W index (which ranges from 0 for no concordance to 1 for perfect concordance). If the two classifiers contained invariant information (i.e., tolerance to irrelevant feature change), the ranking of activations in the two transformed weight maps should be highly concordant. The 95% bias-corrected and accelerated percentile confidence intervals were calculated for different measures.

The edges contributing most to classifier accuracy were then examined. Positive and negative weights in the transformed weight map reflects discrimination supporting the Local and Global level, respectively (due to the coding used in the SVM). The bootstrapped transformed weight maps from the two cross-classification directions were averaged to enhance features relevant to both, and the median of these averages were selected as the most robust weight map summary. The upper and lower 2.5% of the median weights were identified as significant features, and plotted within the original correlation matrix space and projected as graph plots onto the visual field. The space of intra-V1 edges was divided into ten sub-networks as described in Valdes-Sosa et al. (2022). The number of positive and negative edges in each sub-network for the median transformed weight map was counted in all conditions. We then tested if the distribution across sub-networks changed as a function of preprocessing scheme or Level. This produced a three-way contingency table (3 preprocessing schemes x 2 levels x 10 sub-networks) that was analyzed for pattern heterogeneity (that is differences in underlying distributions across multiple contingency tables) using the R package DiffXtables.^8^

### Estimation of eye movements in the fMRI experiment

To estimate potential effects of eye movements on our fMRI data, we employed a post-hoc control utilizing DeepMReye, a convolutional neural network designed for decoding gaze positions from the magnetic resonance signal of the eyeballs (Frey et al., 2021). Specifically, we utilized the variant providing 10 inferred eye positions per scan volume. Since our experiment predated the public release of DeepMReye, no calibration of the network during recording sessions was carried out. Consequently, our analysis was centered on within-subject comparisons across various stimulus conditions. The gaze time series were z-scored within each participant for meaningful comparisons. We assumed that the mean gaze position across all runs closely approximated the central fixation marker. A linear mixed effects model using the following formula was used:


$$\left(X\;\mathrm{or}\;Y\right)\sim \mathrm{Level}*\mathrm{Letter}+\left(\mathrm{Level}*\mathrm{Letter}\;|\;\mathrm{participant}\right)$$

Letter and level were fixed effects and subject a random effect.

### Control eye movement experiment

A control experiment was conducted with 18 additional participants (twelve female and six male, age range 21–61, median = 42) who were Cuban university students or graduates. All had normal (or corrected to normal vision), no history of neuropsychiatric diseases, and 16 were right-handed. Eye movements were measured while the subjects observed the same stimuli -and performed the same task- from the fMRI experiment but with slightly larger stimuli. A 34 × 27 cm monitor screen (1,280 × 1,024 pixels resolution) was used. A chin and forehead rest fixed the participant’s head position at 69 cm from the screen; therefore, the stimuli were about 7.16° wide and 2.9° high. These stimuli were larger in degrees of visual angle than the ones used in the fMRI experiment, thus optimizing the possibility of detecting a stimulus effect on the gaze patterns.

An EyeLink® 1,000 Plus Version 1.0.6 Desktop Mount system (SR Research Ltd., Ontario, Canada) was used to measure eye position by recording corneal reflection and dark pupil with a video-based infrared camera and reflective mirror. These measurements had a spatial resolution of 0.01° of visual angle and a temporal resolution of 1,000 Hz. The viewing was binocular, but the recording was monocular. Calibration and validation of the measurements were performed before each experimental session. The fixations during the 20 s stimulation blocks were separated into sets corresponding to the four stimulus types.

To drive the prediction of stimuli from intra-V1 matrices, eye movement patterns need to be different for each stimulus type and this difference consistent across participants. We tested this possibility by using the iMap4 toolbox (Lao et al., 2017). Fixations for each trial were projected back into their x and y pixel coordinates in the visual field as Dirac deltas with amplitude proportional to their durations. This fixation duration map was convolved with a two-dimensional Gaussian function, producing gaze heatmaps that were downsized in pixels with a scale of 0.25. This procedure was repeated with Gaussian kernels with 5-, 10-, 20-, and 40-pixel standard deviations. The resulting 3D matrices ( Fix , ntrials × 320 × 256) were used as the dependent variable and modeled in a mass univariate linear mixed model (LMM) according to the following equation:


$$\begin{array}{l} \mathrm{Fix}\left(x,y\right)\sim 1+\mathrm{Level}+\mathrm{Letter}+\mathrm{TrialOrder}+\mathrm{Level}:\\ \mathrm{Letter}+\left(1\;|\;\mathrm{participant}\right) \end{array}$$

Level was Global or Local, letter was E or U, and TrialOrder is the order of presentation for each type of stimulus in each participant.

The LMMs at each pixel were fit by maximal likelihood (ML) using the *fitlme* function from the Statistics Toolbox™, Matlab 2022b (MathWorks Inc., MA, USA). Subsequent analysis was in two steps. First, the original parametric statistical values from the LMMs were thresholded at a given *p*-value, which in different iterations was 0.05, 0.1 and 0.2 divided by the number of pixels with non-zero signals (7165) to form clusters. This step was repeated after resampling with replacement across participants (1,000 times). For the original data and the bootstrapped data, the cluster mass was obtained by summing the contrast coefficient values of the LMM within each cluster. An empirical distribution was obtained of these cluster mass measures. The cluster mass of the original clusters was then compared with a bootstrap distribution under the null hypothesis values, accepting as significant clusters in the *p* < 0.05 rightmost tail. This non-parametric procedure allowing to correct for the multiple comparisons inherent to the mass univariate nature of the heatmaps statistical tests.

### Simulation of effect of eye movement in fMRI correlation matrices

Simulated time series, based on the data from the control experiment, were generated to gauge the possible effects of gaze patterns on V1 fMRI activity and thus the classifiers used here (see flowchart in Supplementary material). As in previous work (Kay et al., 2008), V1 was modeled as a pyramid of Gabor filters. The bank of filters consisted of five resolution levels (1, 2, 4, 8, and 16 cycles/FOV), eight orientations (0, 22.5, 45, 67.5, 90,112.5, 135, and 157.5°), and two phases that tiled the screen at evenly spaced positions according to the resolution (2, 8, 16, 64, and 256 respectively). All the spatial resolution levels were included in the subsequent simulation, which probably overestimates the information available in the fMRI.

The stimulus patterns on the screen were convolved with the gaze trajectories for their corresponding blocks for each participant. The two phases and eight orientations from the output from the Gabor filter bank were collapsed at each position for all scales for each fixation episode. The simulated fMRI time series consisted of segments in which the filter output for each fixation was expanded in time according to their durations. The artificial time series were convolved with the canonical HRF function and downsampled to the fMRI TR (2.5 s). In contrast to real data, no noise was added to the artificial time series, to optimize the possibility of detecting the influence of gaze patterns on the classifiers. Correlation matrices (size: number of Gabor-filters x number of Gabor-filters) were calculated with these time series for each stimulus in all participants. The same classification procedures and permutation tests used for the fMRI data were applied to these artificial correlation matrices.

## Results

### Classification results in the main experiment

**Figure 3:**
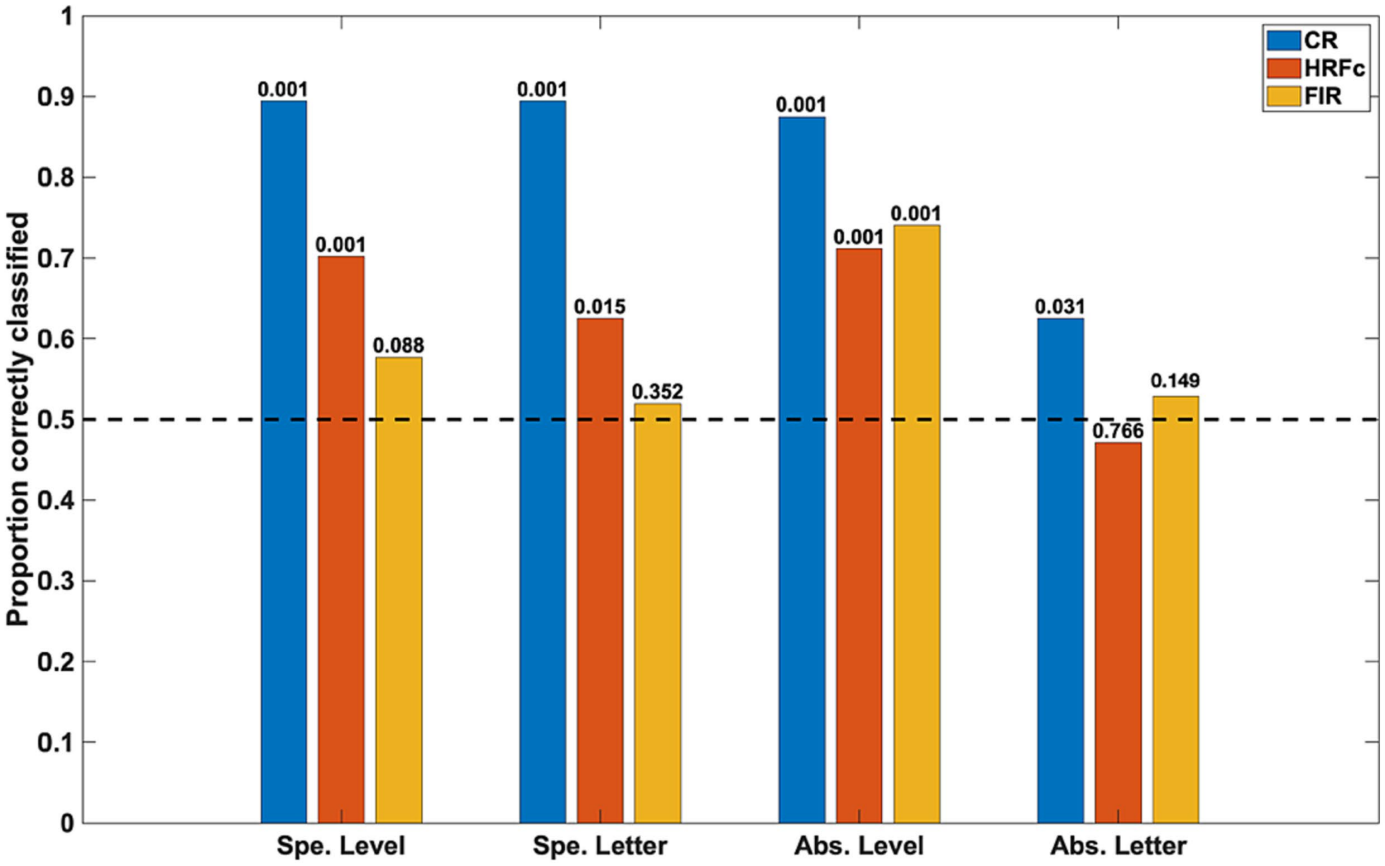
Classification accuracy as a function of stimulus property and preprocessing scheme. The specific (Spe.) classifiers were LOSO (inter-subject). The Abstract (Abs.) case were intra-subject cross-classifications. Significance of rejection of null hypothesis (chance or 0.5 proportion correct) in the permutation test is exhibited above the bars. CR is common response, HRFc is deconvolution with the canonical hemodynamic function, and FIR is deconvolution with finite impulse responses.

The accuracy of the classifiers for the three preprocessing schemes is shown in Figure 3. Inter-subject classifications for specific properties (for level, averaging Global vs. Local for E and for U; for letter, averaging E vs. U for Global and Local) were significant in the permutation tests for the CR and for the signals deconvolved with the canonical HRF, but not for the signals deconvolved with FIRs. This implies stability across subjects of the intra-V1 correlation topologies associated with each stimulus condition except for FIR deconvolution.

The intra-subject invariant cross-classification for Level was significant for all preprocessing schemes, which means that a change in letter identity did not adversely affect this prediction. In contrast, invariant Letter cross-classification was at just above chance for the CR and non-significant for both types of deconvolved signals in the permutation tests. These results indicate that the spatial scale of stimuli (invariant to shape) is well reflected in the topology of intra-V1 networks, whereas letter shape independent from the spatial scale is not. Summarized otherwise, the classifications with the CR matrices were always accurate, with canonical HRF deconvolution classification was also good except for abstract Letter, whereas with FIR deconvolution classification was accurate only for abstract Level. We also examined cross-classifications with a LOSO scheme to verify stability of results across subjects. The results were analogous to the intra-subject case (average accuracy for abstract Level CR = 0.75, HRFc = 0.66, FIR = 0.61; for Letter CR = 0.63, HRFc = 0.55, FIR = 0.50).

### Equivalence of weight map structure from the level cross-classifier

**Figure 4:**
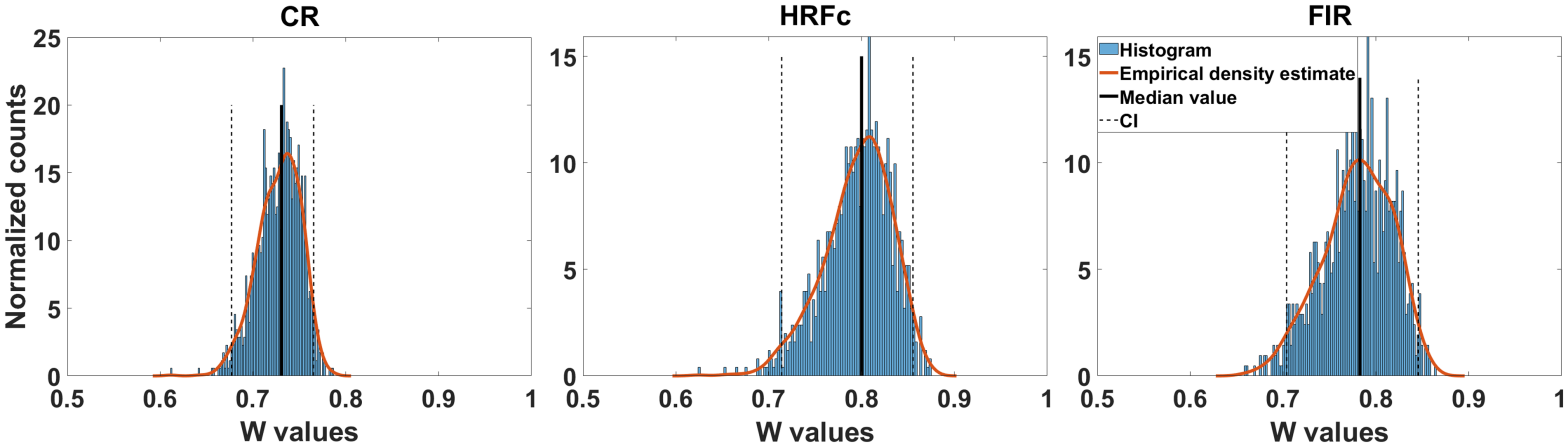
Concordance of feature weight maps in the two directions of abstract Level cross-classification. These directions were U - > E and E- > U. Concordance was measured with Kendall’s W coefficient in repeated classifier training instances with resampling across participants with replacement. For the three types of preprocessing, the bootstrapped confidence intervals were above chance, indicating equivalent structure of the weight maps.

Since accuracy in the cross-classifier for letter identity was at chance, we only show transformed weight maps for abstract Level. The concordance of the weight maps for the Level cross-classifier across the two letter identities was large for all preprocessing schemes (median Kendal W from about 7.5 to 0.8). As seen in Figure 4 the confidence interval is well above chance (0.5). This indicates that the structure of the weights for classifying level are common across the two letters and is congruent with accurate learning transfers between the two instances of level cross-classifiers.

### Topology of weight maps for the abstract level cross-classification

The feature weight maps of abstract Level were examined in more detail to characterize the V1 connections driving the successful discriminations. This classifier was trained repeatedly using sampling with replacement across participants and the intra-V1 connections (i.e., edges of the correlation matrices) that appeared in the 2.5 and 97.5% tails of the empirical distribution of the median bootstrap values were selected as most significant edges.

**Figure 5:**
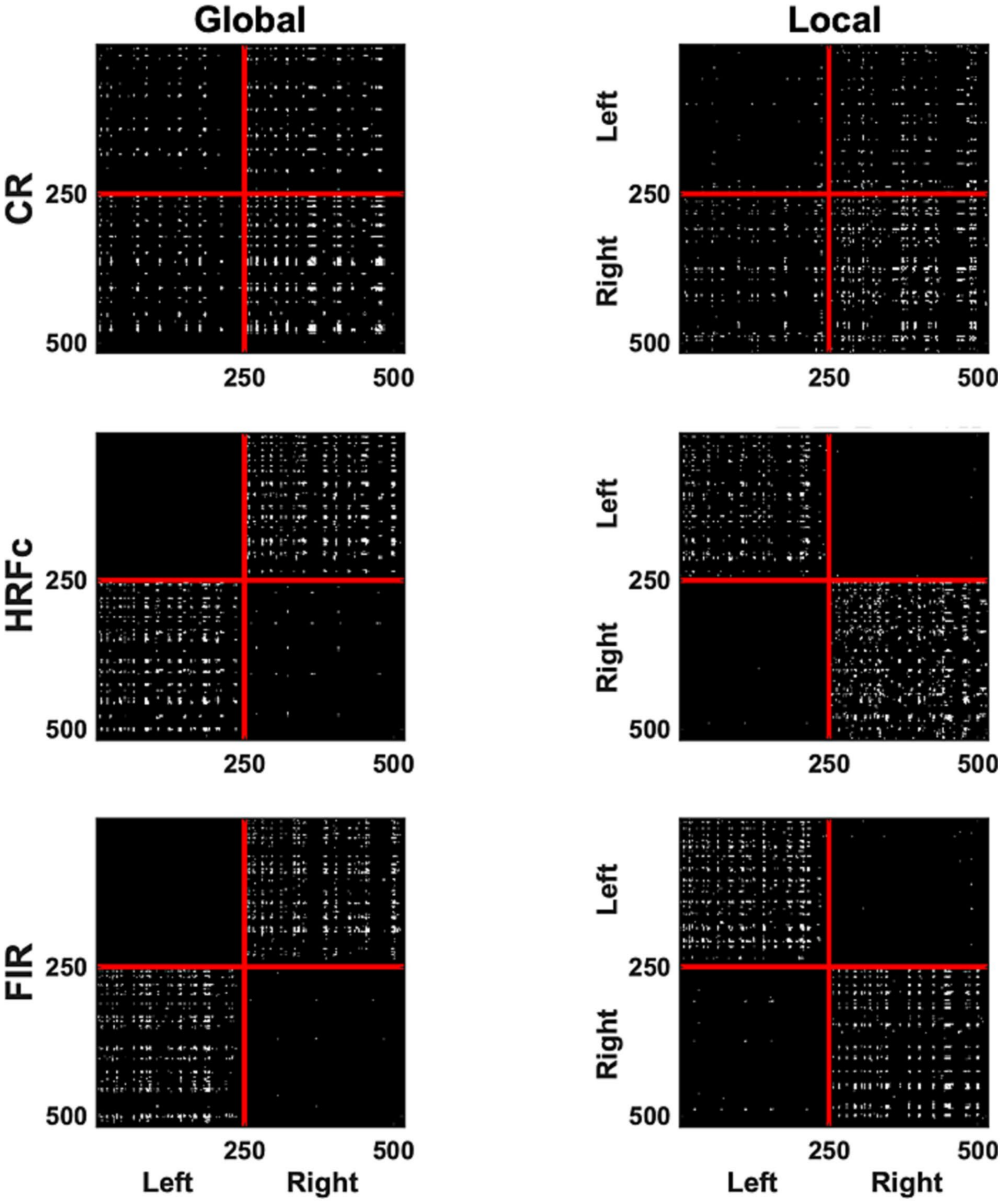
A binary matrix representation of the edges contributing most to abstract Level cross-classification for the three pre-processing methods (acronyms as in Figure 3). Edges were selected if their weights in the classifier were in the top (selecting Local) and lowest (selecting Global) 2.5% of the weight value distribution. In each matrix the left V1 vertices are placed sequentially on the top (y axis) and to the left (x axis) of the plots, and the right hemisphere vertices follow.

The most significant edges (contributing to classification accuracy) can be observed in the representation of V1 correlation matrices in Figure 5. The significant edges with the common response occupy all quadrants, for both levels, although slightly more for right–right connection for the Global, and slightly less for left–left for the Local level. The pattern of results is strikingly different after HRFc and FIR deconvolution. In these matrices, significant edges for the Global level are predominantly interhemispheric (left–left and right–right), whereas intra-hemispheric (right–right and left–left) edges dominate for the Local level. We tested the nonindependence of the distribution counts across quadrants as a function of preprocessing scheme, and if the weights were positive (Local) or negative (Global), with a loglinear model in R. All models were highly significant (*p* < 0.0001), with the best fit for the one including all two-way associations of factors.

**Figure 6:**
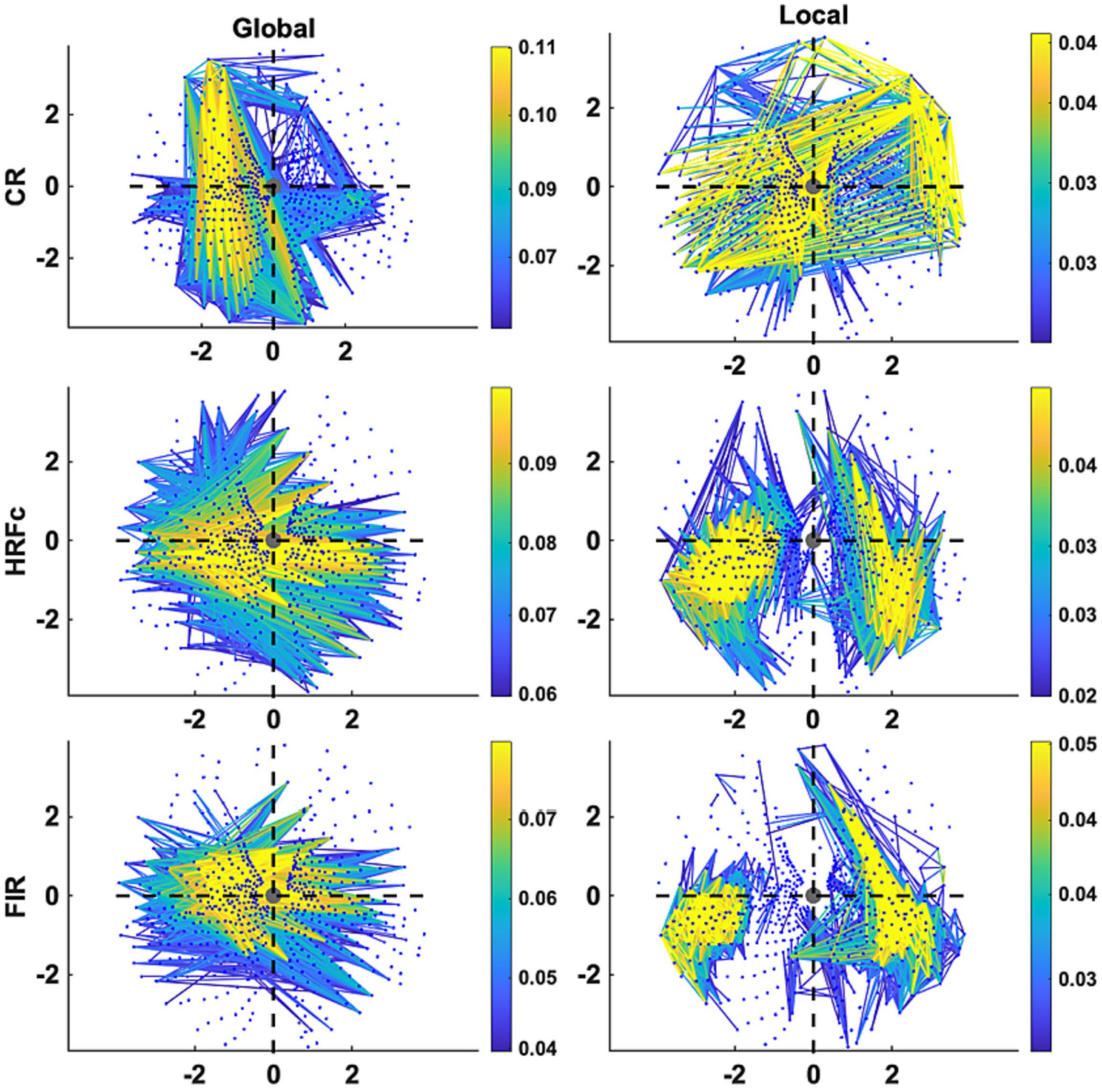
Graph plot representation in the visual field of the edges contributing most to Level cross-classification for the three pre-processing methods (acronyms as in Figure 3). These are the same edges represented in Figure 5. Axis represents degrees of visual angle, and the central grey circle the fixation point. Color represents the weight value.

The Graph plots (Figure 6) confirm the findings of the matrix plots while adding more details of the topology of the connections. For the Common Response, significant links are long and predominate in the up-down direction especially in the left VF but are also present for horizontal and quadrant specific links. Note that these position roughly follows the upright strokes common to the Global U and E (absent in the right VF). In contrast, many long and short links widely spread across the VF are significant for the local condition. For both matrices with deconvolved data, the Global level significant links tended to be inter-hemispheric (linking left and right VFs), both horizontal and diagonal in orientation, with few connections between the upper and lower VFs. Up-down and interquadrant connections dominated the topology of the Local level. HRFc and FIR associated topologies were very similar.

**Figure 7:**
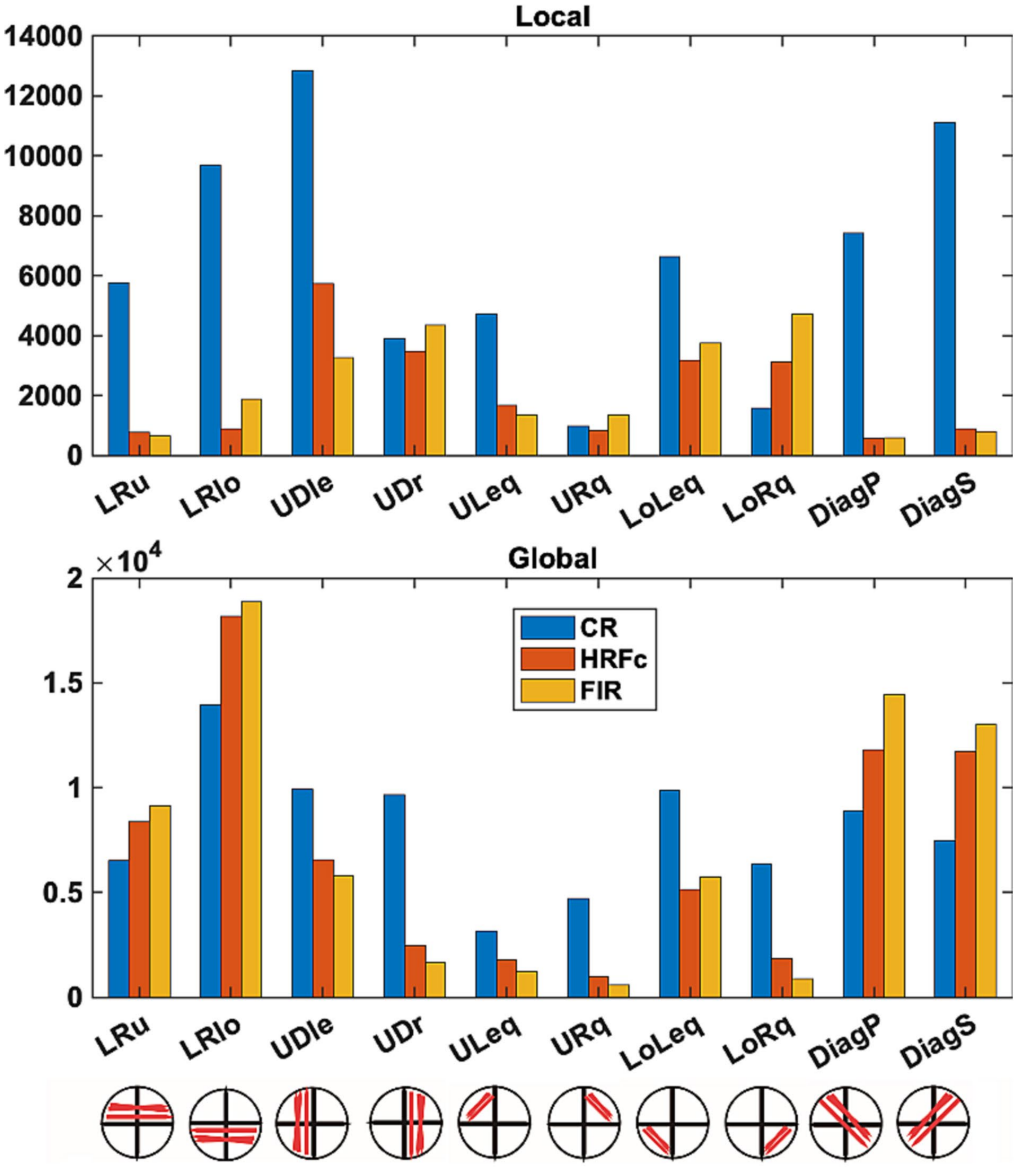
Number of weights favoring each level in intra-V1 sub-networks as a function of preprocessing scheme. Edges of each sign in the intra-V1 correlation matrices were counted within the sub-networks comprising connections in specific directions (see lower insert).

Counts of all positive and negative (favoring Local and Global respectively) weights are shown as a function of sub-network in Figure 7. Differences in underlying distributions across all the multiple contingency tables are very clear and were confirmed by the tests with the DiffXtables package (all comparisons significant at *p*-value <2.2e-16). The relative abundance of weights favouring the Global level is greatest for the lower horizontal VF sub-networks (LRlo), followed by the principal (DiagP) and secondary diagonal (DiagS), and then the lower horizontal sub-network. Interestingly, in these sub-networks the number of counts is largest for the FIR, second largest for HRFc, and smallest for the CR. In other sub-networks there are more counts for CR but very few for the two deconvolved cases. The counts for weights favouring the Local show a very different pattern. For the deconvolved data the most involved sub-networks are the up-down links in both left (UDle) and right (UDr) VFs and the connections limited to the lower quadrants (LoLeq and LoRq). The CR counts are the largest for these weights but present in all sub-networks.

### Analyses of eye movements

To drive the prediction of stimuli from intra-V1 matrices, eye movement patterns need to be different for each stimulus type and consistent across participants. The post-hoc analysis using DeepMReye, failed to reveal any systematic difference in gaze placement (as inferred from the eyeball fMRI signals) between stimulus conditions. All coefficients in the linear mixed effects analysis were small (<0.02) and the associated *t*-tests were non-significant (abs(t) < 1.2).

**Figure 8:**
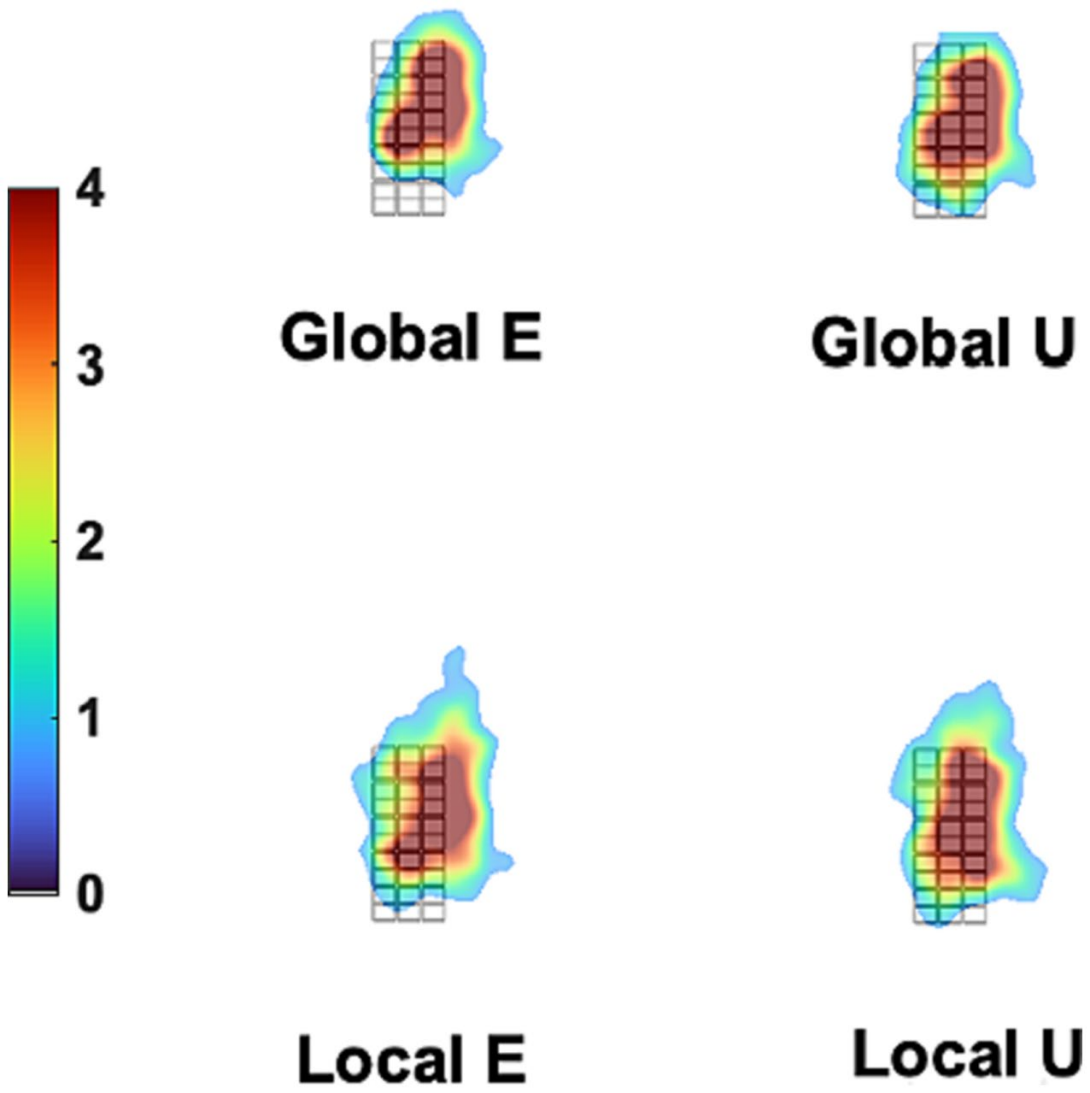
Mean Gaze heatmaps for different stimuli. The grid in black lines is the background stimulus (which defines the dimensions of all other stimuli, see Figure 1), placed at the center of the stimulation screen. The overlaid heatmaps represent the mean density of fixation times were placed at each site of the screen, collapsing trials and participants for each stimulus, as measured in the control experiment. There is very little difference between stimuli in density distribution. This was formally tested with the iMap4 toolbox.

**Figure 9:**
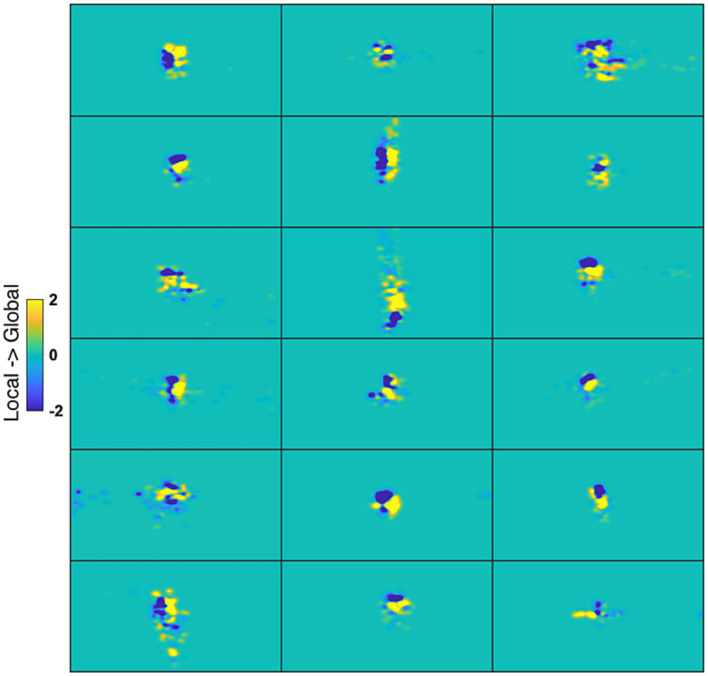
Gaze heatmaps for individual cases. Each panel is a map from one participant representing the difference between Global and Local stimuli (collapsing across letter identity) of the time gaze was spent at each screen point. Blue coloring at a point reflects larger fixation time for Local, and yellow for Global.

Additionally, we tested the possible role of eye movements in a control experiment under conditions that would have exaggerated any between-stimulus difference in gaze patterns. Figure 8 displays the fixation-time heatmaps for the four stimuli used here averaged across participants. The gaze patterns are very similar for all stimuli. Figure 9 shows the difference in fixation-time heatmaps between the Global and Local levels for all the participants. It is obvious that the heatmaps do not replicate across individuals, with different configurations of fixation location preferences for the level contrasts. Similar results were obtained for the letter contrast.

Gaze heat maps are critical here since they reflect cumulative and interactive effects of fixation position and duration that could potentially affect the fMRI time series. Neither Letter nor Level was significant after correction for multiple comparisons with the cluster mass method in the iMap4 LMM analysis. This negative result was obtained for all the smoothing kernels tested. Even with an uncorrected threshold *p*-value of 0.001, Level was significant at only 2 pixels.

### Classification of simulated correlation matrices

**Figure 10:**
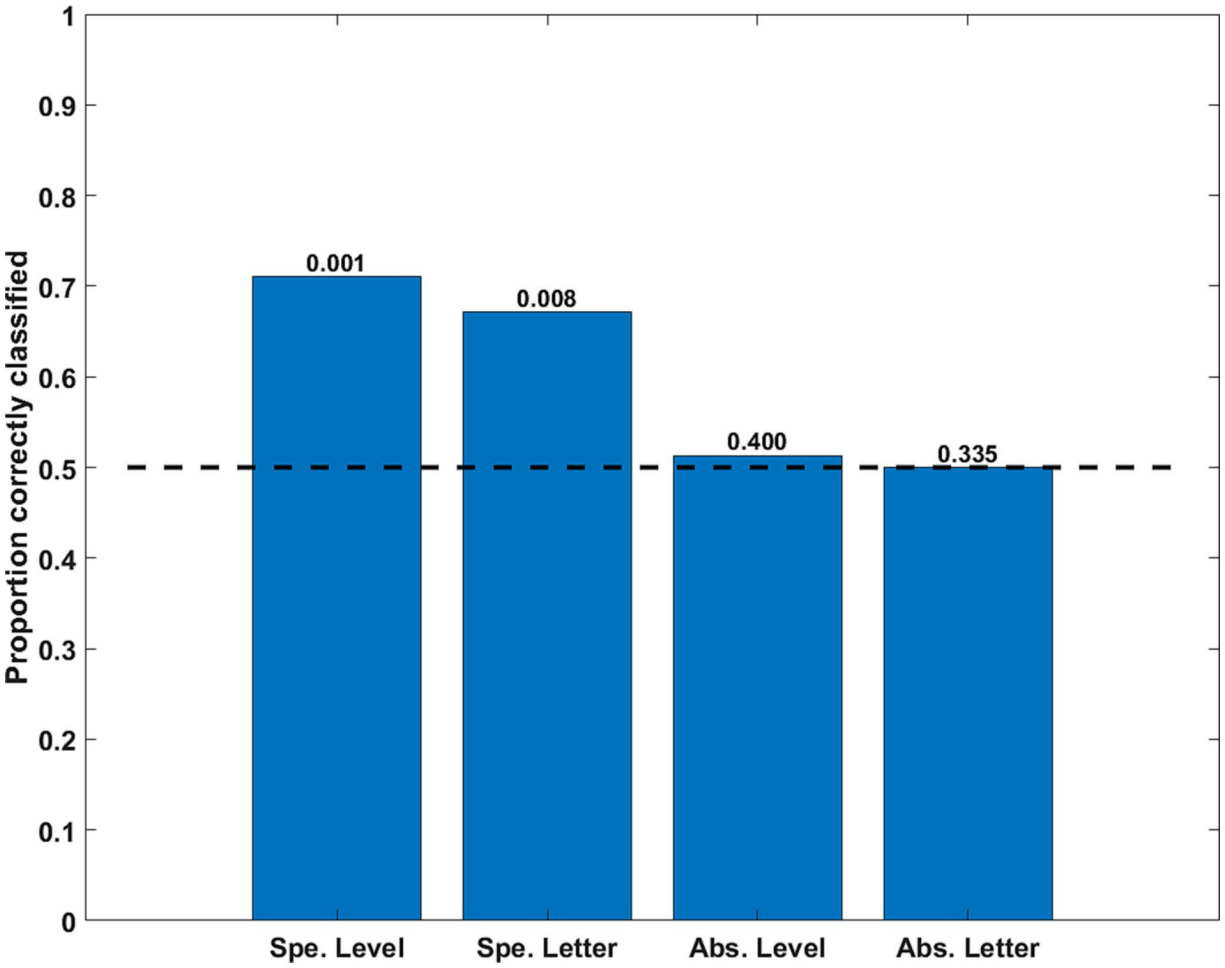
Classification accuracy as a function of stimulus property for the time series simulating eye movement effects. The specific (Spe.) classifiers were LOSO (inter-subject). The Abstract (Abs.) case were intra-subject cross-classifications. Significance of rejection of null hypothesis (chance or 0.5 proportion correct) in the permutation test is exhibited above the bars.

The classification of simple, specific, discriminations was significantly above the chance level. However, both cross-classifications (Level and Letter) were at chance level. Thus, neither the abstract Level (independent from the letter shape) nor the abstract Letter shape was decoded accurately from the simulated correlation matrices (see Figure 10).

## Discussion

Prediction of specific feature conjunctions based on intra-V1 correlation matrices was highly accurate for all fMRI preprocessing methods, but also possible with time series simulating the effects of eye movements on V1 fMRI. More interestingly, cross-classification of stimulus properties was accurate only for Level (ignoring Shape), but failed for Shape (ignoring Level) and for the simulations of eye movement effects. Cross-classification of Level was most accurate for the FIR deconvolved data, then for HRFc deconvolved data, and less so for the common response (CR), although significant in all three. The weight maps for this Level cross-classifier differed between fMRI preprocessing methods, involving distinct V1 sub-networks. The weight maps for deconvolved data suggested that support for the Global over the Local- level mainly involved inter-hemispheric links. In contrast, support for the Local level was found in localized intra-hemispheric links.

Specific classifications were highly accurate in all conditions, which was expected since they would harness all the differences in retinotopic stimulation between stimuli. Since the accuracy of these classifications was equivalent across fMRI preprocessing schemes, both shared stimulus-evoked responses and activity idiosyncratic to individuals could have contributed to the discriminations. The simulations did rule out role for eye movements in this type of classification. We conclude that MVPA with specific stimuli are difficult to interpret. However, the stable association of intra-V1 correlation matrices with stimulus type across individuals found here encourages the search for ways of disentangling the potential sources of the underlying networks.

The most noteworthy finding in this article was that intra-subject cross-classification was accurate for abstract Level (i.e., learning transfer for Level occurred across letters identities) for all preprocessing methods, which contrasts with a failure in cross-classification for letter identity (except for a modest effect with the CR). Also, the feature weight maps for the Level cross-classifiers separately trained on deconvolved data associated to each letter were very concordant with each other. This similarity of weight maps buttresses the idea (Tian and Zalesky, 2021) of a common intra-V1 connectivity topology for Level irrespective of letter shape. Before additional discussion of these results, we examine the possible contribution of eye movement artifacts to V1 synchrony in our experiment.

Post-hoc assessments of eye movements during the fMRI experiment, conducted with the convolutional neural network DeepMReye, did not uncover systematic variations in gaze positions across different stimulus conditions. Acceptable accuracy has been reported with this method in across-subjects decoding schemes with multiple fMRI datasets (Frey et al., 2021). Although this result is probably valid since each subject served as his own control in future studies it would be best to perform intra-experiment calibration of DeepMReye. Systematic variations in gaze positions across different stimulus conditions were also absent in the control experiment. Moreover, the simulations based on this off-line control experiment discourage the idea that fMRI activity associated with eye movements explain the accurate cross-classification of Level described here. Note that these negative results were obtained despite the fact that stimuli in this control were larger than in the original fMRI experiment, a condition that would have optimized detection of different degrees of eye movement between stimulus-types.

The simulated time series were generated from with a Gabor pyramid model with of filters for many spatial resolutions were used. However, real signals are additionally contaminated by noise. Thus, our simulations (together with the larger stimulus size compared to the fMRI experiment) are probably over-optimistic as to potential information for classification. Ramírez and Merriam (2020) rightly point out that decisions about parameters determining the signal to-noise ratio (SNR) of simulated brain patterns in forward models of brain activity, can lead to inconclusive when trying to explain effects in fMRI experiments, especially if the not chosen adequately. Nonetheless, classification of abstract Level was not possible. The small effect of stimulus type on gaze pattern in experiments contradicts a previous study (Sasaki et al., 2001), reporting more bigger eye movements when attending to Global than to Local stimuli. However, the Global stimuli (about 30 DVA) were very large, and the Local stimuli small (about 2.4 DVA), a size ratio of about 13. In contrast, here Global (under 5.3 DVA) and Local (under 1.05 DVA) letters were both small, with a size ratio of about 5, thus confined to the fovea/parafovea.

Since accurate cross-classification of Level was achieved for all types of fMRI preprocessing, both stimulus-evoked responses common to all individuals and idiosyncratic activity could have enabled this discrimination. Deconvolution with canonical HRFs or FIRs are liable to miss-modeling (Lindquist et al., 2009) thus not accounting for all the stimulus-evoked signals. Yet, this procedure undoubtedly reduces the contribution of the common response. Given that the classifier weight maps are very different between the CR and the deconvolved data, we may conclude that distinct neural sources are shaping them. Examination of the graph plots of influential feature weights, and their relative presence in V1 sub-networks, shows that the identification of Global and Local stimuli is based on different network patterns for each type of processing. This means that the common response and subject-idiosyncratic activity both provide information that allows prediction of abstract Level, but they do so via distinct V1 sub-networks.

The weights that support Global stimuli in the Level cross-classifier based on the common response correspond to links that follow the outline of the Global letters within the right VF, where both U and E share upright strokes. The weights favoring the Local level have a broader distribution, in many directions and in many sub-networks, which corresponds with retinotopic layout of the local stimuli. Given that CR reflects the stimulus-evoked response, these patterns could reflect mappings of the feedforward effects of retinal stimulation, although the details of this are not clear.

The weight maps for he cross-classifiers based on deconvolved data indicate that support for Global stimuli came from links crossing the representation of the vertical meridian in V1 and basically those mapping of the lower visual field. This is the congruent with the fact that the Global, but not Local, lettres spanned both visual fields. It is also consistent with evidence that Global visual perception is more accurate in the lower compared to the upper VF (Previc, 1990; Christman, 1993; Levine and McAnany, 2005). This advantage could be explained by greater sensitivity in the lower visual field to lower spatial frequency components (Niebauer and Christman, 1998), which are needed to extract Global shapes, including those necessary to perceive Global Navon figures (Flevaris and Robertson, 2016).

This suggests an interesting hypothesis. Shifting attention towards the Local or Global levels is thought to occur by filtering out higher/lower spatial frequencies from the representation of the retinal input (Flevaris et al., 2011, 2014). When processing Navon figures, the control of spatial scale is essential (Flevaris and Robertson, 2016). Clear differences in spatial frequency spectra (see Iglesias-Fuster et al., 2014) exist between. Our Global and Local stimuli. These differences in spatial frequency could influence the which type of intra-V1 links are attentionally selected, thus driving the weight maps for Level cross-classification described here. In a prior study by our group using activation-based MVPA (Valdés-Sosa et al., 2020), information about Level (independent from shape) was found in the scene-selective cortex (medial ventral occipitotemporal and middle occipital areas). This area could be a source of feedback contributing to the intra-V1 network effect of Level.

Decoding of shape that is tolerant to changes in size is present in the fMRI activations of higher-order-visual areas such as LOC (Grill-Spector, 2003). In our previous study with the same data used here, information about shape invariant to Level was found in activations from these object-selective cortices (Valdés-Sosa et al., 2020). Several hypotheses can be advanced as to why abstract cross-classification for letters in V1 failed here. One is that the fMRI synchrony patterns at the Local level could have been more susceptible to the blurring produced by inter-subject functional misalignment or small eye movements given their small size. Another possibility is that shape-invariant representations do not influence intra-V1 networks through feedback. Although robust functional connections exist between the foveal and parafoveal region of V1 and LOC (e.g., Baldassano et al., 2016), this coupling can be modulated by task requirements (e.g., Al-Aidroos et al., 2012), and perhaps was not present in our experiment. These ideas also require additional testing.

This study has several limitations. Higher-powered replications are needed, given the possibility of false positive results and inflated effect sizes in results from small samples (Button et al., 2013). Another limitation of this study is the lack of calibration of the eye movements measurements during the fMRI recording. Eye movements produce uncontrolled blurring of the retinotopic stimulus representation, which could weaken the correspondence of topologies across participants. An additional limitation is that we used a small range of stimulus shapes. More diverse stimuli (as used in our previous work, Iglesias-Fuster et al., 2014) would allow better testing of V1-connectivity patterns.

We conclude that intra-V1 correlations carry multivariate information about observed Navon letters that is stable across individuals. Furthermore, information of an abstract property, the Level of the stimuli, was present in the intra-V1 correlation matrices. Although eye movements could influence the fMRI to enable decoding of specific stimulus properties, this does not seem to be possible for abstract Level. Stimulus-evoked activity drives part of the synchrony in intraV1 fMRI activity but seems to play a lesser role in the sub-networks that allow abstract classification of stimulus level. These spatial organization of the V1 sub-networks related to abstract level discrimination could mirror asymmetries found in psychophysical studies.

## Data availability statement

The original contributions presented in the study are included in the article/Supplementary material, further inquiries can be directed to the corresponding author.

## Ethics statement

The fMRI study was approved by Ethics committee of UESTC, Chengdu, China. The procedure for the control experiment was approved by the ethics committee of the Cuban Center for Neuroscience. Both studies were conducted in accordance with the local legislation and institutional requirements. The participants provided their written informed consent to participate in these studies.

## Author contributions

MO-O, MV-S, and PV-S: designed research. JP-H and JI-F: collected data and performed an initial data preprocessing. MO-O, MV-S, DM, and PV-S: contributed analytic tools. MO-O and MV-S: analyzed data and wrote the paper. All authors contributed to the article and approved the submitted version.

## Footnotes

^1^ http://www.vislab.ucl.ac.uk/cogent.php

^2^ http://cibsr.stanford.edu/tools/ArtRepair/ArtRepair.htm

^3^ http://www.fil.ion.ucl.ac.uk/spm/

^4^ https://sites.google.com/site/mrilateralventricle/template

^5^ http://surfer.nmr.mgh.harvard.edu

^6^ www.humanconnectome.org/software/workbench-command/

^7^ https://github.com/noahbenson/neuropythy/

^8^ https://cran.r-project.org/web/packages/DiffXTables/index.html
