# Supplementary material for: Intra-V1 functional networks and classification of observed stimuli
Source: Front Neuroinform. 2024 Mar 11;18:1080173. doi: 10.3389/fninf.2024.1080173 (PMC10961393; doi:10.3389/fninf.2024.1080173)
Supplement: Supplementary file 1 [file Data_Sheet_1.docx]

Supplementary material

Description of simulation

The eye tracking data from 18 participants in the control experiment were using to create a simulation of the retinal stimulation changes produced by gaze displacements. Although stimuli were centered at the fixation point, saccades to different pats of the display would produce a relative motion of the stimuli over the retina and thus mapped to different parts of V1. The simulation was created as follows:

1-In all participants, the four stimuli from our experiment were displaced from the fixation point (center of the screen) to the new positions mirroring the changes that the measured saccades would have produced. This is mirror of the real changes in retinal stimulation but since the purpose of this simulation is to see if there is relevant information in elicited neural activity this is sufficient. This data was generated for each stimulus block in the control experiment (identical to the fMRI experiment) and segments for each stimulus concatenated to produce 4 time series.

2- We assumed a linear change in neural response with changes in retinal illumination at each cortical site. The pattern of retinal stimulation from Step-1 passed through a Gabor filter pyramid tiling the visual space occupied by the stimulation screen. This pyramid had a bank of filters consisted of five resolution levels (1, 2, 4, 8, and 16 cycles/FOV), eight orientations (0, 22.5, 45, 67.5, 90,112.5, 135, and 157.5°), and two phases that tiled the screen at evenly spaced positions according to the resolution (2, 8, 16, 64, and 256 respectively).

4-The output of the filter were 5456 time series (one per Gabor filetr) was then fed into the next step.

5-The output from all the filters were convolved in the time domain with the canonical HRF to model the effects of the hemodynamic delay, and downsampled to the fMRI TR (2.5 s). No noise was added to the artificial time series. We were not attempting to make a realistic simulation. Instead we posed an ideal situation to optimize the extraction of information from the gaze patterns.


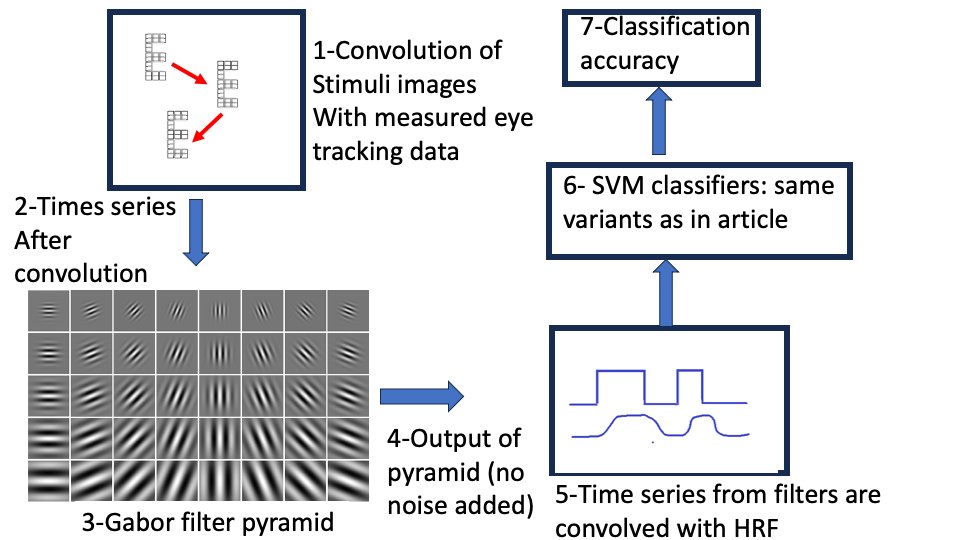
6- These HRF-convolved time series from all the filters were then fed used to train SVM with the same strategies in the article.

7- Permutation tests (shuffling data in the training and testing set) were performed on the artificial data to examine the reliability of the classification results.

Supplementary Figure 1.

Supplementary figure
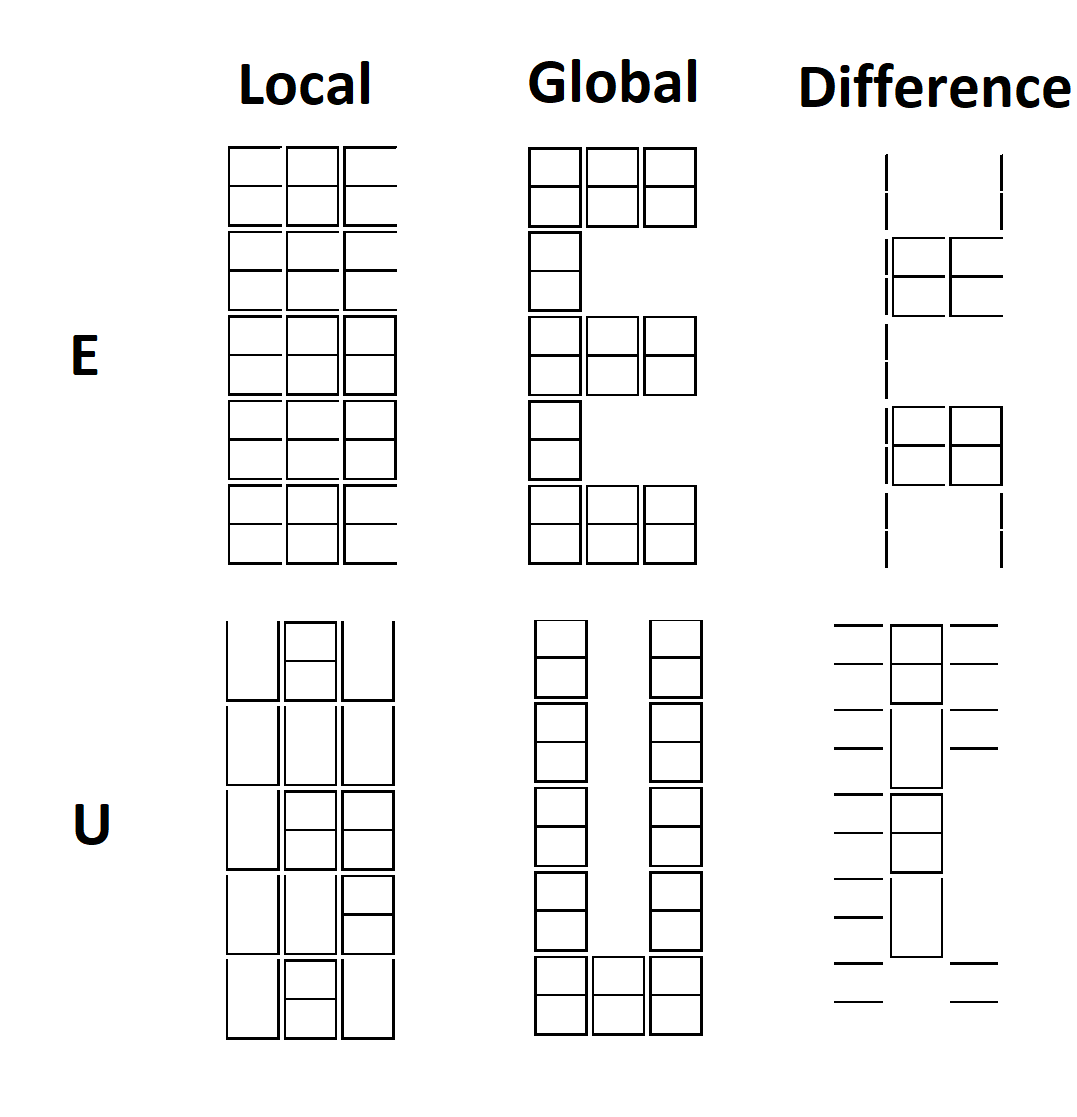
2A

In the first two columns we show the Local and Global stimuli. The last column shows the subtraction of Global and Local, images for the two letters thus the difference in retinotopic stimulation that are present for these stimuli. In the next figure the difference of the two differences. How**ever,** transforms of the patterns of retinotopic stimulation could achieve this equivalence. One transform would be a spatial frequency decomposition of the images, the other the extraction of ore abstract representations of the strokes present in the letters.


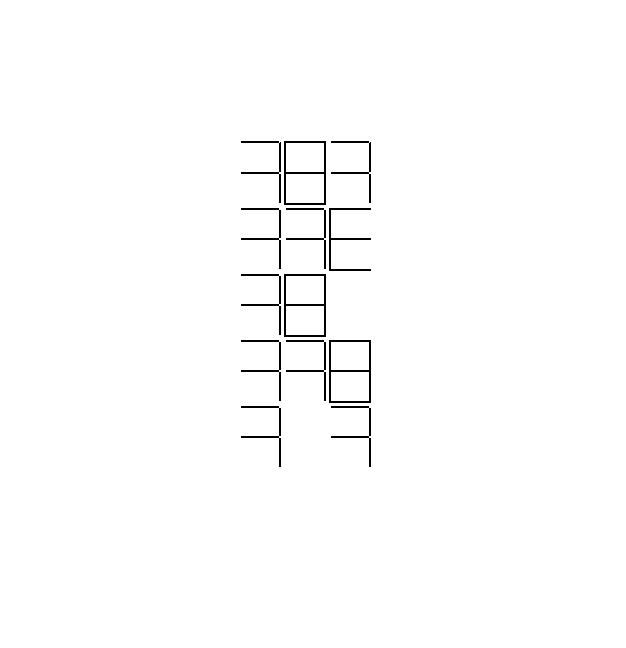
Supplementary figure 2 B

It is obvious from this figure that the direct discrepancies in retinotopic stimulation between levels for the two letters are very different. The pattern of retinotopic stimulation cannot be driving the cross-classifier weight maps because then for the Global/Level discrimination they would be different, and we showed in the article that they are equivalent (concordant).
